# Mr. Franks, on the Brunonian System

**Published:** 1799-12

**Authors:** 


### *To the Editors of the Medical and Phyſical Journal.*

GENTLEMEN,

YOU did me the favour to inſert in the ſecond number of the Medical and Phyſical Journal, ſome Obſervations on the Brunonian ſyſtem of medicine; which were made in conſequence of reading an article in the firſt Number of this Journal, extracted from an eſſay written by Profeſſor HUFELAND, of Jena, intitled, "Remarks on the Influence of the Brunonian Syſtem on the Practice of Medicine." Since that time a very valuable work has engaged my attention; it is a Treatiſe on Febrile Diſeaſes, by Doctor WILSON, Phyſician to the County Hoſpital at Wincheſter. In this work are ſome obſervations on the above ſyſtem. It will be obſerved, that Dr. WILSON is an advocate for this ſyſtem of medicine, although he differs in opinion with the author, in ſeveral particulars.

I could wiſh to offer ſome farther remarks on this ſubject; for either my perceptions of the Brunonian doctrine are incorrect, or Dr. WILSON has charged the author of it with ſome inconſiſtencies, of which he may be acquitted.

---

### SECTION II. page 460 of the above TREATISE.

"OF THE BRUNONIAN DOCTRINE."

"Of the medical ſyſtems which have been propoſed to the world, BROWN's alone is in a great meaſure founded on obſervation; many parts of it are ſimple deductions from facts, which muſt be admitted, independent of all hypotheſis. In a more advanced ſtate of our ſcience, when the ſyſtems of STAHL, HOFFMAN, BOERHAAVE, and CULLEN are forgotten, there will ſtill remain enough of the Brunonian doctrines to preſerve the memory of their Author."

 This

This quotation proves that Dr. Wilson is friendly to the general plan of the doctrine. I ſhall now ſtate ſome parts of it where material differences of opinion are found to occur.

OF THE EXCITABILITY.

Page 473.—" One of the moſt ſtriking inconſiſtencies in the writings of Dr. Brown is his ſuppoſition, that every living body, at the commencement of its exiſtence, receives a certain quantity of excitability, which, if not extinguiſhed by violent ſtimuli, or by too great an abſtraction of ſtimuli, will laſt for a certain length of time. The quantity received (he ſuppoſes) determines the natural duration of life, it being impoſſible to protract it after that quantity is exhauſted."

I have very little to offer with reſpect to the eſſence of excitability, it being one of thoſe things which muſt ever remain above the reach of human comprehenſion. Yet I think, that Dr. Brown's ideas of the vital principle, founded on excitability, aſſiſts us in accounting for the different phœnomena which attend the living ſtate, better than any other ſuppoſition with which I am acquainted.

Such, however, is the imperfect ſtate of human knowledge, that it is poſſible his ſuppoſition—that every living body, at the commencement of its exiſtence, receives a certain quantity of excitability which determines the duration of life—may be erroneous; but whether this ſuppoſition be juſt or erroneous, is not of importance with regard to the general principle or practice. Whilſt life remains, the property which diſtinguiſhes it from dead matter exiſts in the ſyſtem, and will be forced into action, if excitement can be effected; for, the only difference between apparent and poſitive death conſiſts in the preſence or abſence of excitability.

" We know not," Dr. Brown obſerves, " what excitability is, or in what manner it is effected by the exciting powers. But whatever it be, whether a quality or a ſubſtance, a certain portion is aſſigned to every being upon the commencement of its living ſtate. The quantity or energy is different in different animals, and in the ſame animal at different times."

OF EXCITEMENT.

Page 506.—" By excitement," Dr. Wilson obſerves, " is not neceſſarily implied contraction, becauſe the term is not confined to the muſcular ſyſtem; the nervous ſyſtem, however, cannot be excited, without occaſioning a correſponding change in the muſcular."

The

The means uſed for the recovery of perſons apparently dead from ſubmerſion, ſerve to ſhew that it is the muſcular fibres of the heart and arterial ſyſtem, which muſt firſt be excited to enſure ſucceſs. When excitement is produced in this ſyſtem, which may be conſidered as muſcular, then a correſponding change is occaſioned in the nervous ſyſtem, and *ſenſation* returns.

The blood having loſt its ſtimulating effect, in conſequence of the communication with the air of the atmoſphere being interrupted, is the immediate cauſe of death after ſubmerſion; for, according to Dr. Crawford's experiments, the heat contained in arterial blood, compared with the heat in venous blood, is as 1.0300 to .8928;—the left auricle and ventricle having been accuſtomed to a ſtimulus derived from an external agent equal to 1.0300 will not contract upon the application of a ſtimulus only equal to .8928, and poſitive death will be the conſequence, unleſs we, by inflation of the lungs, ſucceed in reſtoring the loſt degrees of ſtimulus, before the excitability is too far withdrawn.

If by excitement, contraction is not neceſſarily implied, it muſt be included in the effect, contraction being a principal and conſtant effect produced during life by a ſtimulus in the blood, and regulated by the degrees of it; for according to the degree is the excitability inherent in the muſcular coat of the arteries *forced* to make regular or healthy contractions, or irregular and unhealthy.

Dr. Wilson does not approve of Dr. Brown's definition of excitement, page 516.—" I do not adopt Dr. Brown's definition of excitement, that it is the effect of agents on the living ſolid, becauſe they often produce a ſtate very different from excitement—Atony."

Dr. Brown's ſyſtem of Medicine is remarkable for the ſimplicity of diſtinctions and terms of ſcience; the diſtinctions apply to practice, and the new terms, which are few in number, convey clear ideas.

The imperfect ſtate in which this author found the ſcience, made it abſolutely neceſſary for him to employ terms which were not till then in uſe, and which I think are unexceptionable.

The diviſion of univerſal diſeaſes (diſeaſes of the ſyſtem) is allowed to be correct, and I think the words which he has uſed to mark the diſtinction here made, could not have been more appropriate,—Sthenic, Aſthenic.

Dr.

Dr. Wilson defines excitement to be "a ſtate of activity, and atony a ſtate of inactivity; that is, of debility, &c."

I do not agree with Dr. Wilson, that the exciting powers produce different ſtates, therefore do not ſee any neceſſity to contraſt the words atony and excitement.

If Dr. Brown, to expreſs a weak ſtimulant, as water, &c. had employed the word *ſedative*, by contraſting the terms *ſtimulant* and *ſedative*, a falſe idea would have been inculcated; therefore it is preferable to reject the term ſedative, and to ſay that water, although a very weak, is ſtill a ſtimulant.

"Diſtilled ſpirits," Dr. Wilson obſerves, page 479, "received into the ſtomach, occaſion excitement; and, to a certain extent, the greater the quantity, the greater the excitement. But the immediate effect of a large quantity of diſtilled ſpirits, ſuddenly received into the ſtomach, has often been inſtant death. Is this excitement?"

There does not appear any thing favourable to the ſuppoſition of atony from this argument. In theſe caſes, a degree of ſtimulant power is ſuddenly applied, which is found to be incompatible with the excitability, and, on that account, life is extinguiſhed—were it compatible with life, the term excitement would be allowed by Dr. Wilson.

The electric fluid is a ſtimulant of the moſt powerful kind; the greateſt degree of excitement, conſiſtent with life, may be communicated by this agent. But ſuch a degree of ſtimulus may be ſuddenly derived from the electric fluid as to put a period to excitement, becauſe the excitability is totally deſtroyed by the ſudden and violent ſhock, to which it is unequal, and which it could not ſuſtain for a moment.

From conſiderations of this kind, I think that the term atony, when oppoſed to excitement, either conveys an erroneous idea, or is without a ſignification.

[ To be Continued. ]

